# Virtual Instrument for Emissions Measurement of Internal Combustion Engines

**DOI:** 10.1155/2016/9459516

**Published:** 2016-03-13

**Authors:** Armando Pérez, Rogelio Ramos, Gisela Montero, Marcos Coronado, Conrado García, Rubén Pérez

**Affiliations:** Engineering Institute, Autonomous University of Baja California, Boulevard Benito Juarez, Insurgentes Este, 21280 Mexicali, BC, Mexico

## Abstract

The gases emissions measurement systems in internal combustion engines are strict and expensive nowadays. For this reason, a virtual instrument was developed to measure the combustion emissions from an internal combustion diesel engine, running with diesel-biodiesel mixtures. This software is called virtual instrument for emissions measurement (VIEM), and it was developed in the platform of LabVIEW 2010® virtual programming. VIEM works with sensors connected to a signal conditioning system, and a data acquisition system is used as interface for a computer in order to measure and monitor in real time the emissions of O_2_, NO, CO, SO_2_, and CO_2_ gases. This paper shows the results of the VIEM programming, the integrated circuits diagrams used for the signal conditioning of sensors, and the sensors characterization of O_2_, NO, CO, SO_2_, and CO_2_. VIEM is a low-cost instrument and is simple and easy to use. Besides, it is scalable, making it flexible and defined by the user.

## 1. Introduction

Air pollution is caused in large part by vehicles due to the burning of fossil fuels that emit pollutant gases such as NO, CO, SO_2_, and CO_2_ [1, 2]. Diesel has been one of the most used fuels in internal combustion engines for more than one century [3]. It is due to its high availability, competitive prices, and high energy density. Diesel is used, for example, in urban transport, cargo transport, light-duty vehicles, agricultural machinery, ships, and electricity generation. Emissions generated by diesel combustion directly affect the health and population life quality. Likewise, these are largely responsible for the climate change problem [4–6].

The biodiesel utilization is an alternative to reduce the pollutant emissions from vehicles with internal combustion engines that run on diesel. Biodiesel is a renewable and environmental-friendly fuel that is derived from lipids. These react with a short chain alcohol in the presence of a catalyst, no matter whether it is acid, basic, or enzymatic, producing a monoalkyl esters mixture of fatty acids [7, 8]. The physicochemical characteristics of biodiesel are similar to diesel. Therefore it may be replaced partially or completely, since the currently applied technology does not require major changes for its use.

Biodiesel has become more important worldwide, and it has shown rapidly industrial growth as an alternative fuel instead of diesel [9]. Therefore, it is necessary to measure the respective emissions to determine the emissions decrease regarding the fossil fuels.

International governments and organizations are currently introducing new regulations that establish more strict emission limits to reduce the greenhouse gases emissions [10–12]. As a result, it is essential to have efficient systems that measure the emissions from internal combustion engines, as well as regulate them.

(*1) Current Systems for Emissions Measurement of Internal Combustion Gases*. The measurement systems for emissions measurement of internal combustion usually use specialized analyzers that work under standardized methods [13]. These provide related information about the composition and quantity of the combustion gases. It is a useful tool to understand and regulate combustion. The implementation of these systems is based on taking a gas sample that is produced in, for example, boilers, engines, and industrial ovens. The sample passes through the electrochemical cells analyzer, obtaining the concentration of each of its components and defining its own quality. In this way, if it is carried out with the relevant current regulations is established, at the same time ensuring the equipment is in proper conditions with its resulting fuel saving.

There are several types of systems in the market for emissions measurement of internal combustion; these may also be fixed or portable depending on their implementation. Conventional systems have several disadvantages, among them we emphasize the high costs purchase from US $2.000 to $20.000, strictness, and impossibility of being scalable [14, 15]. It means that these systems may be only used for certain applications, without having the facility and flexibility to adapt them to other required uses. Currently, the systems based on the virtual instrument have been used as an alternative to the conventional systems for emissions measurement.

(*2) Virtual Instrument Used for the Emissions Measurement of Internal Combustion Gases*. A virtual instrument is a concept that includes software and hardware systems, using a computer, that it replaces a measurement instrument and monitoring in the real world. Every piece of software and hardware that performs this function is called virtual instrument (VI). When a commercial system is used, in most of the cases the virtual instrument concept is performed in an object-oriented programming language [16]. The new scientific instrument supports the innovation and development of systems based on virtual instrument. The virtual instrument advantages are the enclosed adaptability that is included in the software and hardware and the reduction of cost for acquisition channel compared to the conventional instruments of rigid hardware of which function is determined by the manufacturer and also the easiness to be customized accordingly to the specific needs of each user and the programming language use [17]. Virtual instruments integrate nonexclusive operation hardware and powerful software, obtaining as a consequence an instrument of a scalability architecture. This means it may be modified if it is required [18–20].

The virtual instrument implementation has been recently suggested for emissions measurement of internal combustion gases [21]. It is due that the measurement is currently performed by using stand-alone modular analyzers, used, and specialized, providing information about the gases emitted from motor combustion. Hence, virtual systems have been developed to measure and monitor the CO concentration in the gases emitted by vehicles [22].

National Instruments developed a virtual instrument for the emissions measurement generated by internal combustion engines. This instrument is based on the international emissions standards, in particular, the Euro 4 and EPA. These agencies specify the total amount of pollutants that an internal combustion engine must emit to the atmosphere. These emission factors units are defined in general as gram per mile [23].

In this work, a virtual instrument for the measurement and monitoring of emissions (VIEM), based on the LabVIEW 2010 virtual programming platform, was developed. VIEM is synchronized with the sensors, data acquisition device, and signal conditioners to measure and register in real time the emissions of O_2_, NO, CO, SO_2_, and CO_2_. The VIEM programming, the electronic schematics diagrams used for the signal conditioning sensors, and the sensors characterization of O_2_, NO, CO, SO_2_, and CO_2_ are presented as results [24].

## 2. Hardware System Configuration (Hardware Details)

**Figure 1:**
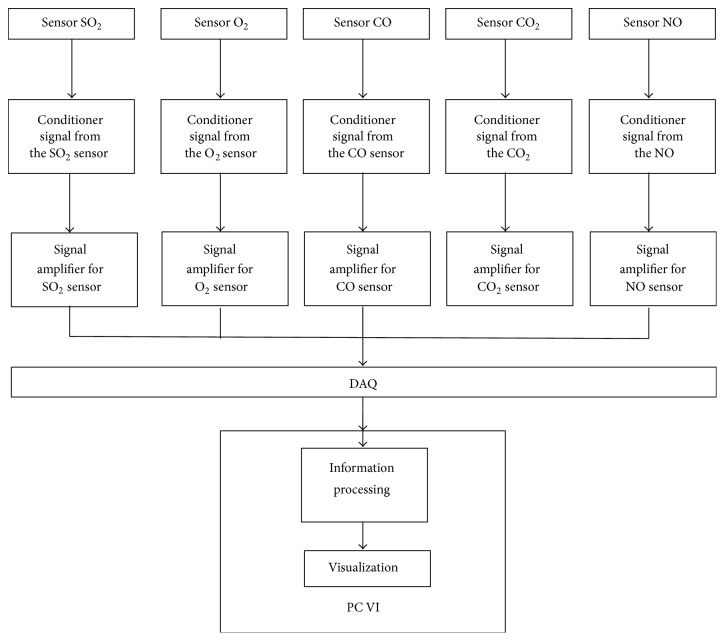
Operating block diagram of VIEM.

Four main devices were involved in the system hardware configuration: firstly, the O_2_, NO, CO, SO_2_, and CO_2_ sensors, the characteristics and operating ranges for each of which are shown in Table 1; secondly, the signal conditioners and amplifiers for each sensor; thirdly, the data acquisition device (DAQ); and, lastly, the personal computer that runs the virtual instrument software, as is shown in Figure 1.

The DAQ digitizes the information that signal conditioners transmit. A DAQ board USB 6009 National Instruments model was used. Digital signals are transmitted to the laptop computer Sony Vaio, VGN-CR190 Intel Core Duo T7100 @ 1.8 GHz model, using Windows 7 operation system.

### 2.1. Infrared Sensor

The infrared sensor works on the basis that is known as the nondispersive infrared analyzer (NDIR), and it is used to detect the presence of carbon dioxide to a volume of 100%. It is possible to determine the CO_2_ concentration using an infrared source with a specific filter, in which it is installed in the optical-gas cavity. The infrared sensor is connected to an electronic signal. Signals process involves the linearization and compensation in the temperature, using algorithms in the system software [13].

### 2.2. Infrared Sensor Operation

**Figure 2:**
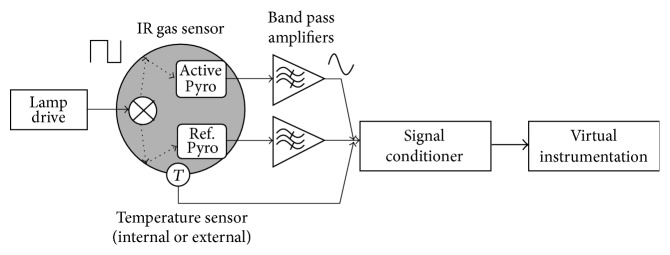
Blocks diagram of a typical gas detector system using an infrared sensor.

The infrared gas sensor uses a low-frequency flash lamp drive that is controlled by an excitation circuit. Infrared radiation pulses reflect inside providing a trajectory through the gas and objective. “Pyros” pyroelectric detectors are used to determine the infrared signal change. The active pyroelectric is sensitive to the changes in the infrared wavelengths that are usually absorbed by the gas, passing between the transmitter and receiver.  Figure 2 shows the block diagram of signal conditioner circuit or line amplifier.

### 2.3. Electrochemical Sensor Operation

**Figure 3:**
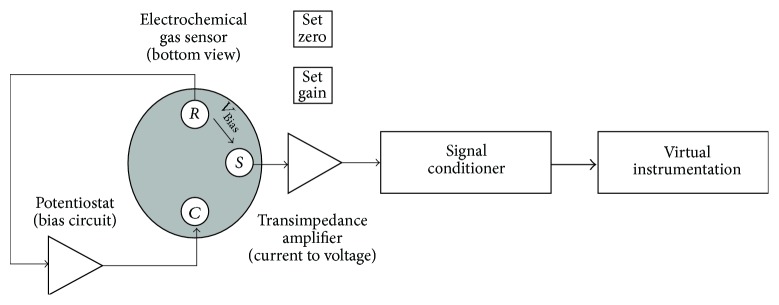
Block diagram of a typical gas detection system.

Electrochemical sensors use a solid catalytic electrolyte making electrons flow of a gas cell sample to a gas cell reference possible. In practice, catalytic coated ceramic materials, for example, ZrO_2_, remove the reference cell that contains a high concentration of O_2_, as well as an electric sample. When the temperature increases, the electrolyte enables the oxygen ionic components transfer from the reference cell to the sample cell. The surface of the electrolyte has a special electrocatalyst layer catalyzing the transfer processing, and it works as an electrode to attract the released electrons. The ions that move from the reference side to the lateral release electrons of the sample on this surface are forced into the equilibrium. However, since the sample is replenished continuously, an ongoing electrons flow is induced through the measurement load resistance. Thereafter, the electric current is used to deduce the oxygen concentration of the electric gas sample [13], as is depicted in Figure 3.

**Figure 4:**
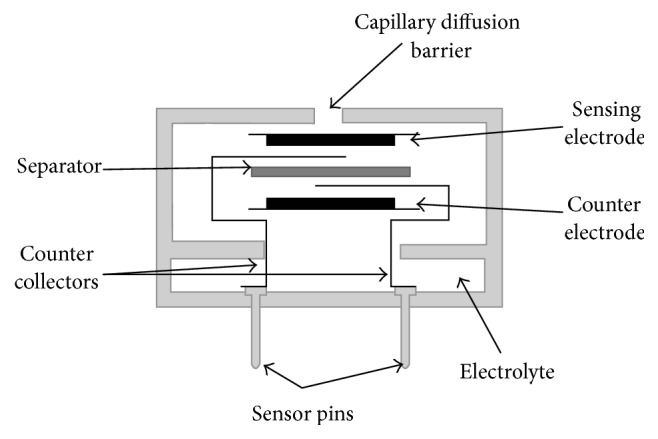
Internal sensor structure of CO_2_.

Because the electrons contain a finite catalytic activity, it is necessary to establish a limit to the diffusion speed of objective gas in the sensor, guaranteeing that gas reacts properly. It is performed through a barrier taking a shape of small hole or capillary located on the sensor cover, as is shown in Figure 4.

### 2.4. Integrated Circuits

Integrated circuits are used for conditioning and amplifying the signal that the sensors send when the amount information or gas concentration is registered. Registered voltage *V*_*o*_ is of 2.435 V when showing 1% of oxygen; this is the minimum concentration that sensor can detect. It means that data acquisition device (DAQ) processes increments that are equivalent to 0.065 V/1% of O_2_. Equation (1) was used to calculate the output voltage:

(1) $$\begin{array}{c} V_{o}=2.5\;V-65\;mV/\%*\left[\;O_{2}\%\;\right]. \end{array}$$

**Figure 5:**
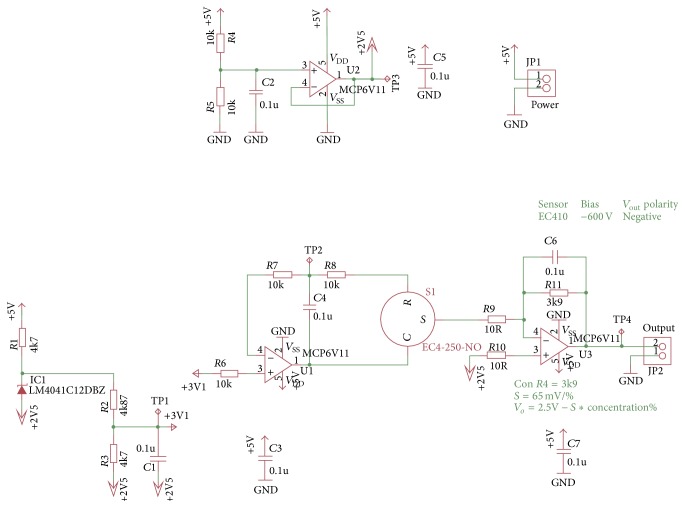
Circuit designed for O_2_ Conditioning signal.

Figure 5 shows the integrated circuit diagram used for conditioning and amplifying the signal provided by the O_2_ sensor.

Registered voltage *V*_*o*_ is of 2.508 V when showing 1 ppm of NO; this is the minimum concentration that sensor can detect. It means that DAQ processes increments that are equivalent to 0.004 V/1 ppm of NO. Equation (2) was used to calculate the output voltage:

(2) $$\begin{array}{c} V_{o}=2.5\;V+8\;mV/ppm*\left[\;NO\;ppm\;\right]. \end{array}$$

**Figure 6:**
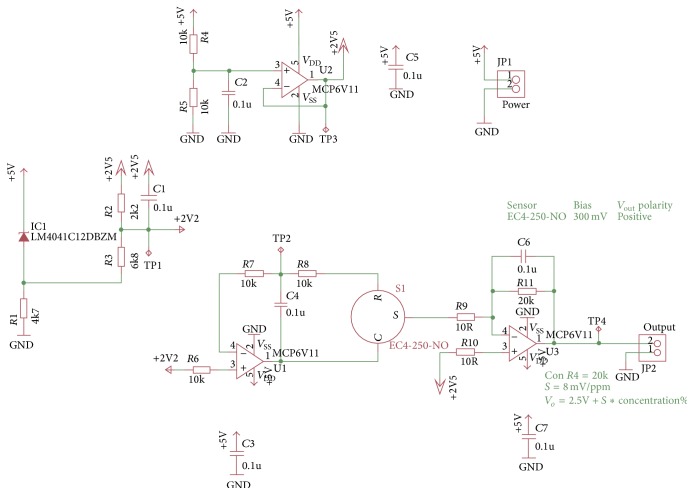
Circuit designed for NO signal conditioning.

Figure 6 shows the integrated circuit scheme used for conditioning and amplifying the signal provided by the NO sensor.

Registered voltage *V*_*o*_ is of 2.5047 V when showing 5 ppm of SO_2_; this is the minimum concentration that sensor is able to detect. It means that DAQ processes increments that are equivalent to 0.00476 V/1% of O_2_. Equation (3) was used to calculate the output voltage:

(3) $$\begin{array}{c} V_{o}=2.5\;V+0.952\;mV/ppm*\left[\;{ppm\;SO}_{2}\;\right]. \end{array}$$

**Figure 7:**
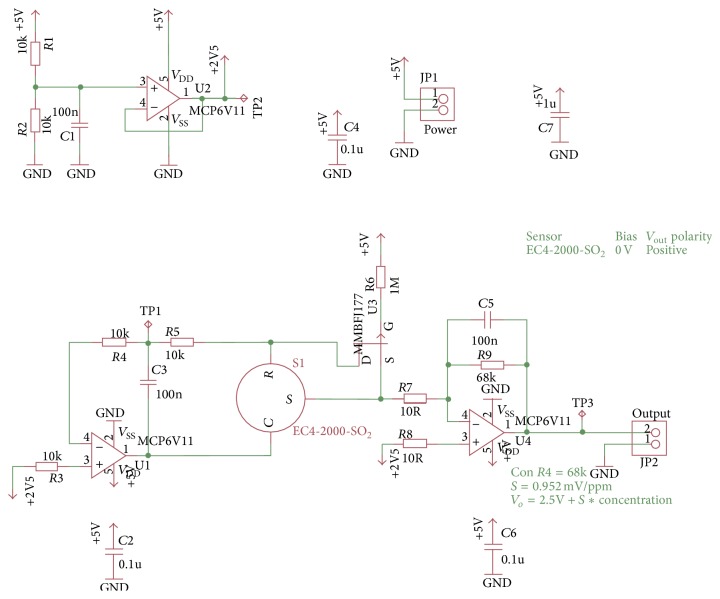
Circuit designed for SO_2_ signal conditioning.

Figure 7 shows the integrated circuit scheme used for conditioning and amplifying the signal provided by the SO_2_ sensor.

Registered voltage *V*_*o*_ is of 2.5039 V when showing 1 ppm of CO; this is the minimum concentration that sensor can detect. It means that the DAQ processes increments that are equivalent to 0.00392 V/1 ppm of CO. Equation (4) was used to calculate the output voltage:

(4) $$\begin{array}{c} V_{o}=2.5\;V+3.92\;mV/ppm*\left[\;CO\;ppm\;\right]. \end{array}$$

**Figure 8:**
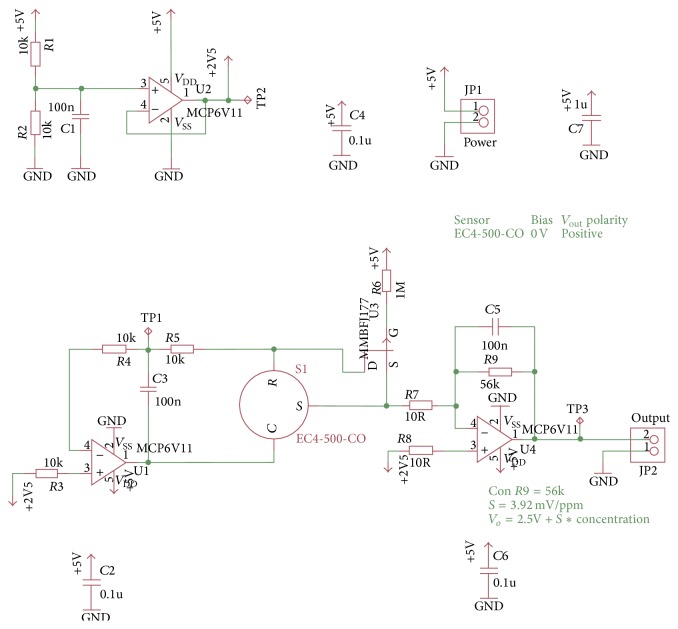
Circuit designed for CO conditioning signal.

Figure 8 shows the integrated circuit scheme used for conditioning and amplifying the signal provided by the CO sensor.

**Figure 9:**
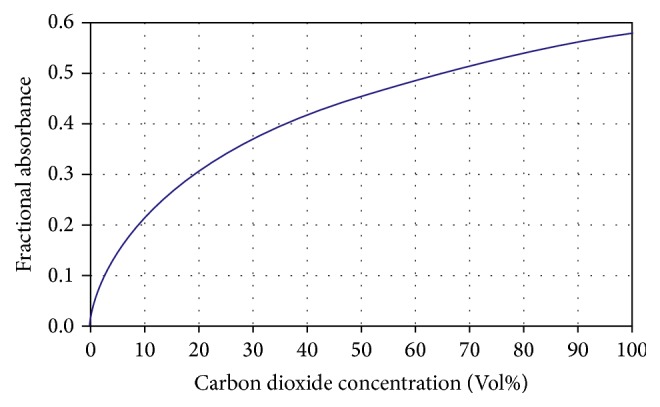
Absorbance fractional versus CO_2_ concentration.

Figure 9 shows the absorbance fractional reaction regarding CO_2_ concentration. It allowed observing the sensitivity of the sensor that oscillates between 0 and 100% of the volume concentration of carbon dioxide.

**Figure 10:**
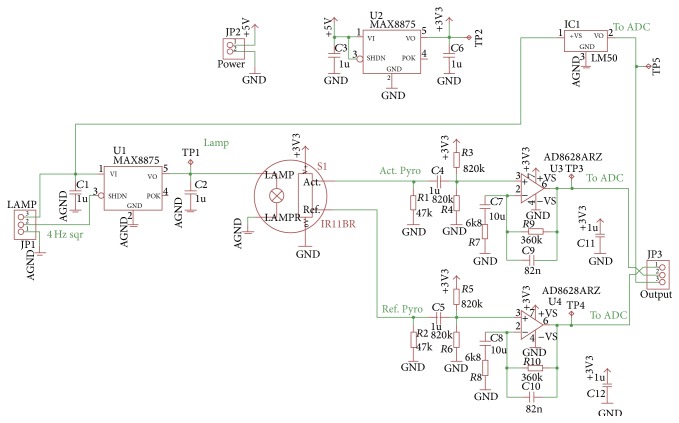
Circuit designed for CO_2_ signal conditioning.

Figure 10 displays the integrated circuit scheme used for conditioning and amplifying the signal provided by the CO_2_ sensor.

## 3. Software Programming for Virtual Instrumentation Development

This section shows the programing description of the main blocks that are part of the virtual instrument for emissions measurement that was developed in LabVIEW 2010 platform. In the figures, the operation performed by each programing block is shown.

### 3.1. Test Counter

**Figure 11:**
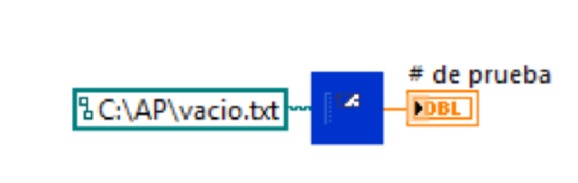
Test counter programming block.

The VI counts the tests that have been performed to organize the files of the experiment. It is performed through the sub-VI test counter, which it saves in a text file (.txt) with a value that starts at zero and increases to 1 for each VIEM run. The corresponding programming block is shown in Figure 11.

### 3.2. Configuration Window

**Figure 12:**
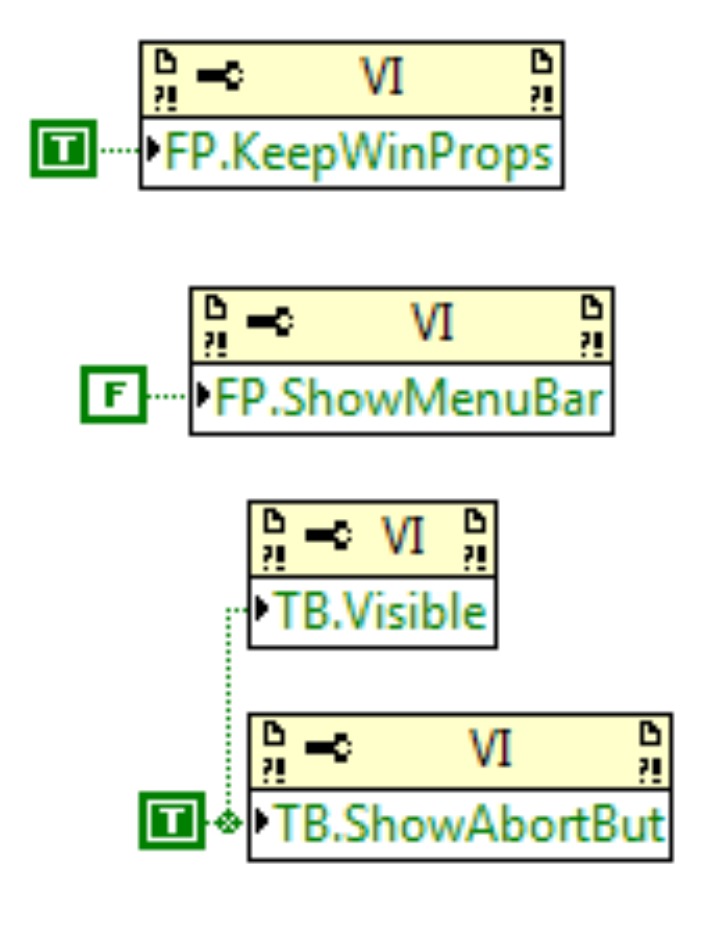
Programming block of the configuration window.

The VI configures the set menus and toolbars using a property-node; these will be shown during the VIEM running. In Figure 12 is observed the programming block of the configuration window.

### 3.3. While Loop 1

**Figure 13:**
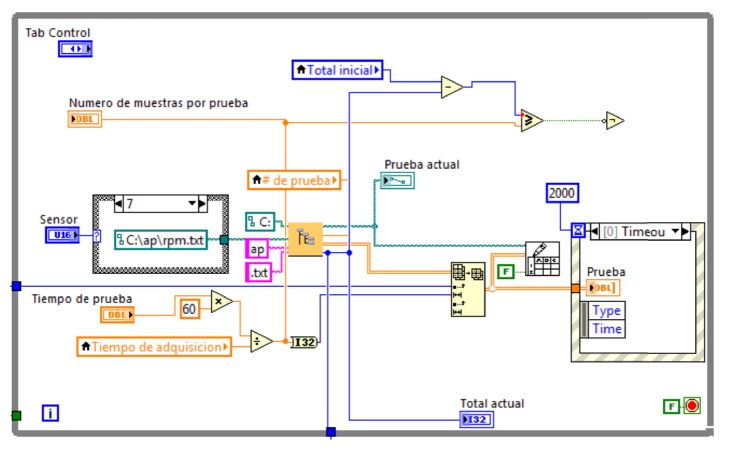
While loop 1 programming block.

While loop 1 runs routines for the configuration and test running. The length of the test is selected using the testing time control. The sample rate configuration is performed using an acquisition time control. The sub-VI “direction by testing” creates the direction and file name containing the samples of test that is in running process. The function sub-VI “Write to Spreadsheet File” creates a new file for each registered variable; this will be shown using a “testing” graphic indicator. The abovementioned is found in the “Event Structure” module, which it enables to synchronize with the computer clock using a time out of 2.00 ms. While loop 1 keeps in ongoing running. In Figure 13, the programming block of while loop 1 is exposed.

### 3.4. While Loop 2

**Figure 14:**
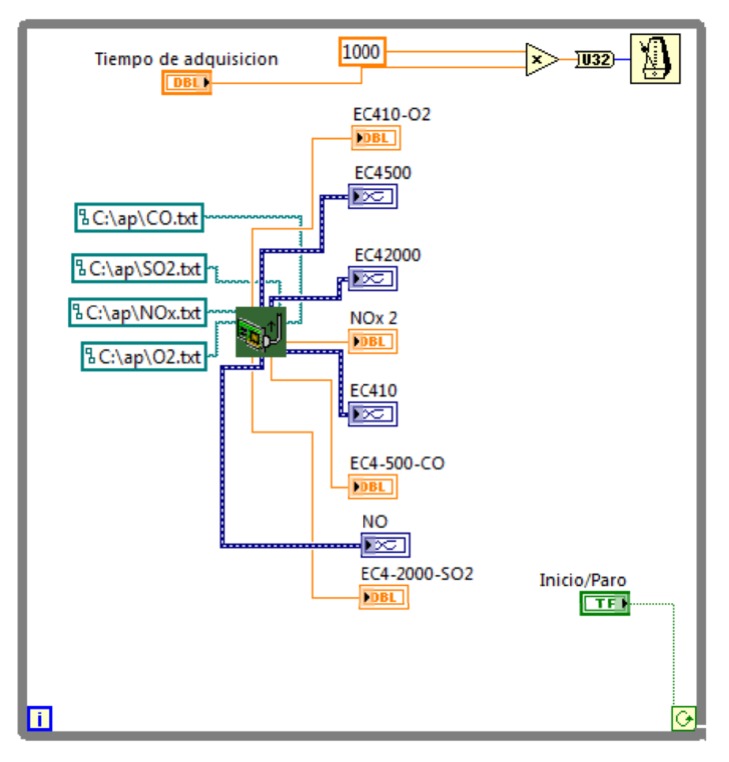
While loop 2 programing block.

While loop 2 is used to receive the sensor signals. It is possible using sub-VI “acquisition.” Likewise, it configures the storage inputs directions for different variables of measurement displaying them through numerical indicators of “double” type. This series frequency depends directly on the configured value in the “acquisition time” control. This series has a run of “continue if true” type that is controlled using the run/stop button.  Figure 14 presents the programming block of while loop 2.

### 3.5. Sub-VI Acquisition

**Figure 15:**
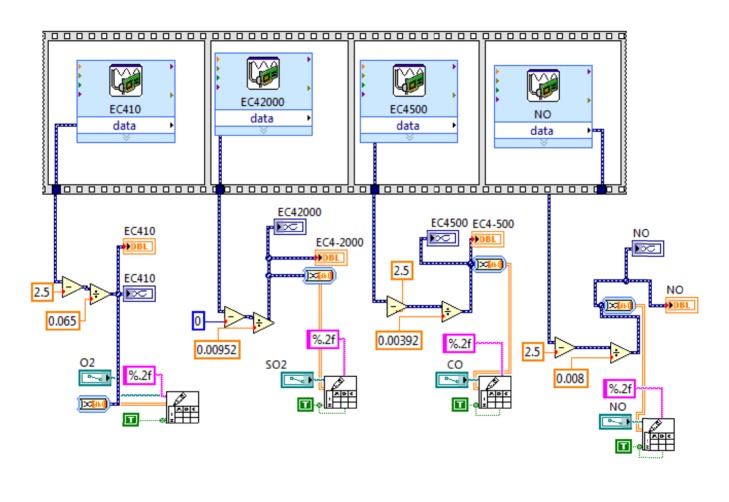
Sub-VI acquisition programing block.

Sub-VI acquisition is used for data collection of the sensors using a DAQ ASSISTANT Express VI, by which it is configured as a, nondifferential analog input to a voltage range from −10 to 10 volts. The virtual instrument “acquisition” output shows to the user the readings in a numeral indicator. Data are processed for its storage using a function sub-VI Write to Spreadsheet. It is performed for a maximum amount of 8 sensors, and data flow is monitored using the “Flats sequence” structure to avoid overload information in the acquisition time.  Figure 15 reveals the sub-VI acquisition programming block.

**Figure 16:**
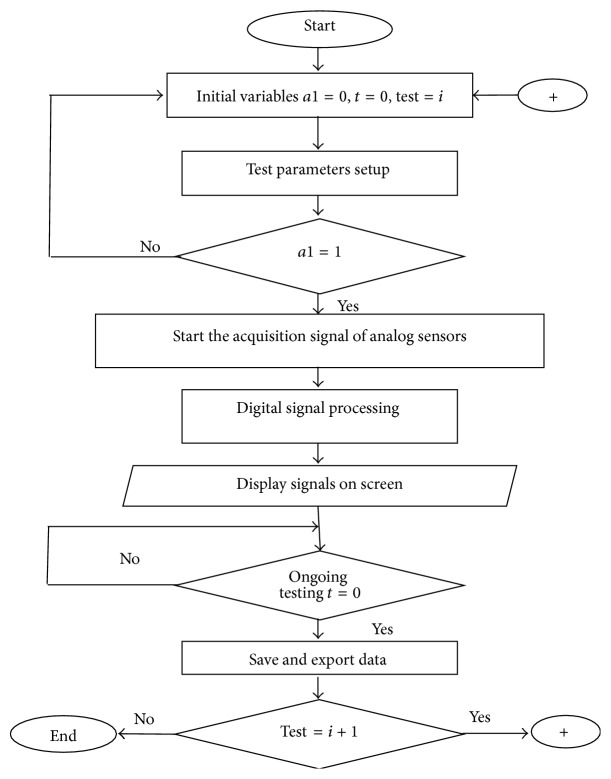
Operation flow scheme of VIEM.

Graphical programming in LabVIEW 2010 uses the programming model for data flow in contrast to the programming based on text, basing its programming in the flow control model. Instead of typing complicated text lines, risking making syntactic and logistics mistakes, LabVIEW 2010 is based on the icons connections with wires using. In addition, for most users, including the advanced users it is easier to view and read the graphical programming compared with the text programming.  Figure 16 illustrates the operation flow diagram of VIEM.

## 4. Applications

The main operation of the measurement system for gas emissions of internal combustion is to measure the gases concentration of O_2_, NO, CO, SO_2_, and CO_2_, for which it uses electrochemical and infrared sensors. Sensors measure the emissions that are generated from a diesel engine that runs with diesel-biodiesel blends. The measurement is performed once the combustion gases are conducted through a pipeline. The flow is firstly conducted to an oil separator and finally to a heat exchanger. Then, the gases are conducted to an exhaust manifold, where the different outputs are connected to the sensors to meter readings in real time, register and storage data provided by the signal conditioners devices and processed through the virtual instrument in the PC. There is the versatility to meter readings every 15 and 20 seconds, as well as 1 and 5 minutes, to create a database.

Other virtual instrument applications are used in monitoring stations, chemical stations, steel industries, restaurants, power stations, combined cycle, internal combustion diesel engines, gasoline, natural gas, and departments of motor testing vehicle. They represent an option for the utilization of the VIEM through its redesign and adaptation to work as a portable system.

**Figure 17:**
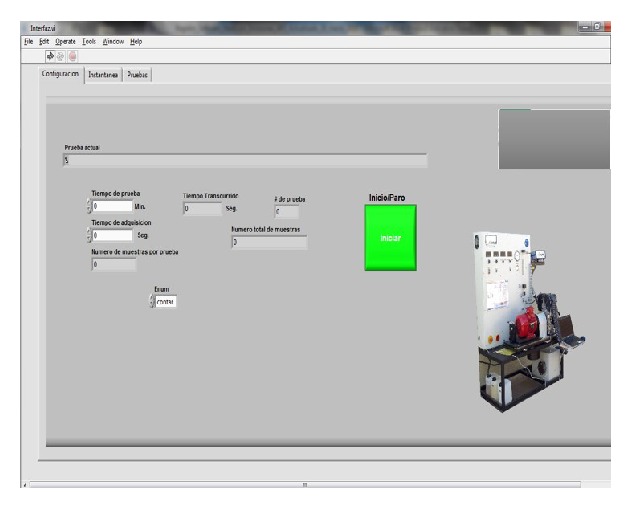
Main window graphical user interface, tab settings.

Figure 17 shows the graphical interface that virtual instrument (VIEM) provides at the moment to select the configuration option. The software enables determining the testing time, the acquisition data time, and the option to start the established tests.

**Figure 18:**
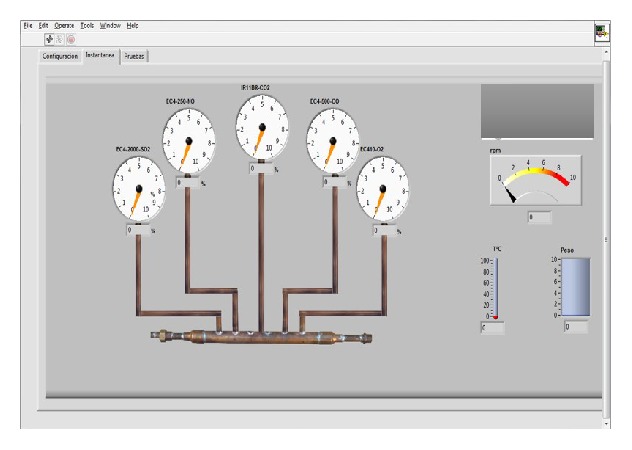
Graphical user interface, instant tab.

Figure 18 shows the graphical user interface, where measurements can be seen in real time of O_2_, NO, CO, SO_2_, and CO_2_ gases, temperature, fuel consumption, and revolutions per minute (rpm).

**Figure 19:**
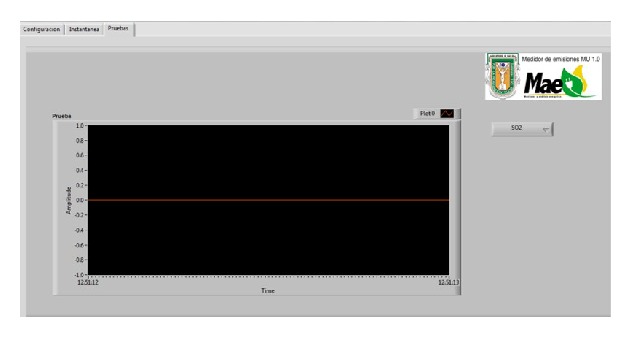
Graphical user interface, testing tab.

Figure 19 highlights the emissions measurement, temperature, rpm, and fuel consumption results. The user is able to select the running results to plot them in a graph.

## 5. Conclusions

In this work a virtual instrument based on the LabVIEW 2010 platform was developed to measure and monitor the emissions such as O_2_, NO, CO, SO_2_, and CO_2_ from internal combustion engines that run on gasoline, diesel, or diesel-biodiesel blends. It is an open architecture instrument and user-friendly. It is possible to modify its graphical interface and include new operations, for example, sensors that measure other emissions depending on the type of concentration and pollutant volumes, according to the user needs.

The VIEM is a useful tool for the emissions measurement that could be used by the industry sector and motor vehicles, as well as its use in the research and academics.

It is recommended to add more sensors to the VIEM to measure a wider range of gases as well as some sensor or analyzer to measure in the order of parts per million.

## Figures and Tables

**Table 1:** Sensors characteristics and operating ranges.

Features	Operating conditions				
	SO_2_	NO	O_2_	CO	CO_2_
Operating ranges	0–2000 ppm	0–250 ppm	0–30% O_2_	0–500 ppm	0–100% Vol.
Model	EC4-2000-SO_2 _	EC4-250-NO	EC410	EC4-500-CO	IR11BR
Sensitivity	8–20 nA/ppm SO_2_	320–480 nA/ppm NO	N/A	55–85 nA/ppm CO	N/A
Maximum overload	N/A	1000 ppm	100% O_2_	1500 ppm CO	N/A
Zero in air at 20°C	<±50 ppm SO_2_	−0.06 To 4.5 ppm NO	N/A	<±3.1 ppm of CO	N/A
Zero deviation (−20 to +40°C)	0 to 4 ppm SO_2_	N/A	N/A	N/A	N/A
Resolution	5 ppm SO_2_	0.5 ppm NO	0.1% O_2_	1 ppm CO	N/A
Response time	*t*90 < 60 s	*t*90 = 35 s	*t*90 < 15 s	*t*90 < 30 s	N/A
Temperature range	−20 to +50°C	−20 to +50°C	−20 to +50°C	−20 to +50°C	−20 to +50°C
Operating humidity	15–90% RH noncondensing	15–90% RH noncondensing	15–95% RH	15–90% RH noncondensing	0–99% noncondensing
Pressure range	90–110 kPa	90–110 kPa	90–110 kPa	90–110 kPa	80–120 kPa.
DC supply recommended	N/A	N/A	N/A	N/A	5 V
